# Effect of H_2_ treatment in a mouse model of rheumatoid arthritis‐associated interstitial lung disease

**DOI:** 10.1111/jcmm.14603

**Published:** 2019-08-19

**Authors:** Yasuhiro Terasaki, Mika Terasaki, Satoshi Kanazawa, Nariaki Kokuho, Hirokazu Urushiyama, Yusuke Kajimoto, Shinobu Kunugi, Motoyo Maruyama, Toshio Akimoto, Yoko Miura, Tsutomu Igarashi, Ikuroh Ohsawa, Akira Shimizu

**Affiliations:** ^1^ Department of Analytic Human Pathology, Graduate School of Medicine Nippon Medical School Tokyo Japan; ^2^ Department of Molecular and Cellular Biology Nagoya City University Nagoya Japan; ^3^ Division of Laboratory Animal Science, Graduate School of Medicine Nippon Medical School Tokyo Japan; ^4^ Department of Ophthalmology, Graduate School of Medicine Nippon Medical School Tokyo Japan; ^5^ Biological Process of Aging Tokyo Metropolitan Institute of Gerontology Tokyo Japan

**Keywords:** D1CC transgenic mice, molecular hydrogen, reactive oxygen species, rheumatoid arthritis‐associated interstitial lung disease

## Abstract

Rheumatoid arthritis (RA)‐associated interstitial lung disease (ILD), a primary cause of mortality in patients with RA, has limited treatment options. A previously established RA model in D1CC transgenic mice aberrantly expressed major histocompatibility complex class II genes in joints, developing collagen II‐induced polyarthritis and anti‐cyclic citrullinated peptide antibodies and interstitial pneumonitis, similar to those in humans. Molecular hydrogen (H_2_) is an efficient antioxidant that permeates cell membranes and alleviates the reactive oxygen species‐induced injury implicated in RA pathogenesis. We used D1CC mice to analyse chronic lung fibrosis development and evaluate H_2_ treatment effects. We injected D1CC mice with type II collagen and supplied them with H_2_‐rich or control water until analysis. Increased serum surfactant protein D values and lung densities images were observed 10 months after injection. Inflammation was patchy within the perilymphatic stromal area, with increased 8‐hydroxy‐2ʹ‐deoxyguanosine‐positive cell numbers and tumour necrosis factor‐α, BAX, transforming growth factor‐β, interleukin‐6 and soluble collagen levels in the lungs. Inflammatory and fibrotic changes developed diffusely within the perilymphatic stromal area, as observed in humans. H_2_ treatment decreased these effects in the lungs. Thus, this model is valuable for studying the effects of H_2_ treatment and chronic interstitial pneumonia pathophysiology in humans. H_2_ appears to protect against RA‐ILD by alleviating oxidative stress.

## 1 INTRODUCTION

In most patients, rheumatoid arthritis (RA) is characterized by increased rheumatoid factor and anti‐cyclic citrullinated peptide antibody levels, which can be detected using commercially available assays.^1^, ^2^ The aetiology of RA may involve the combination of a specific genetic background (certain major histocompatibility complex class II molecules) and environmental triggers, such as infection and smoking,^3^, ^4^ causing defects in immune regulation and inflammatory mechanisms associated with oxidative stress‐induced joint tissue damage.^1^, ^4^ The main manifestation of RA is joint disease; however, recent estimates suggest that more than half of all patients with RA develop some form of extra‐articular lung disease.^5^ In addition, approximately 30% of patients with RA with lung involvement develop RA‐associated interstitial lung disease (RA‐ILD), a serious diffuse parenchymal lung disease associated with impaired gas exchange and fibrotic injury of alveolar septa. The most common histological patterns of RA‐ILD are usual and non‐specific interstitial pneumonia,^6^ which are the primary causes of morbidity and mortality in such patients 7; however, the pathogenesis of RA‐ILD is unclear, and treatment options are limited.

We previously established a model of arthritis‐prone D1CC mice to provide a basis for the development of therapeutic interventions for RA‐ILD. This novel mouse RA model is transgenic for the DBA/1 background. The model uses a class II transactivator and collagen type II (CII) promoter and features aberrant major histocompatibility complex class II gene expression in the joints.^8^

The administration of a low CII concentration to D1CC mice, which are highly susceptible to arthritogenic stimuli, induces the gradual development of chronic inflammatory arthritis. This contrasts with the conventional mouse model of collagen‐induced arthritis, which is characterized by acute inflammation. D1CC mice exhibit interstitial pneumonitis, in addition to RA‐like synovitis with pannus formation and joint destruction, and develop anti‐cyclic citrullinated peptide antibodies.^8^ Thus, we expected D1CC mice to serve as a model that is considerably similar to RA‐ILD in humans and function as an important investigative tool to model human RA‐ILD and chronic interstitial pneumonia. However, to date, we have only obtained data on lung histology 6 months after CII injection in D1CC mice using elastica and Kernechtrot staining.^8^

Autoreactive T cells that infiltrate the synovial tissue in RA promote an immune response and result in the overproduction of pro‐inflammatory cytokines such as tumour necrosis factor‐α (TNF‐α) and interleukin‐6 (IL‐6). Thus, therapy in early RA targets aggressive biological disease alteration by regulating synovial T cells and decreasing the levels of cytokines associated with the disease.^9^ Current treatment options for RA‐ILD include disease‐modifying anti‐rheumatic drugs and biological anti‐TNF‐α therapies. However, evidence suggests that although these treatments benefit joint disease, they can exacerbate pulmonary dysfunction as a side effect.^10^

In addition to these immunogenic factors, reactive oxygen species (ROS) are important therapeutic targets because they occur upstream of cytokine‐mediated inflammatory cascades.^11^ The synovial fluid and peripheral blood of patients with RA contain high levels of ROS and ROS‐induced molecules such as superoxide, hydroxyl radicals (•OH) and peroxynitrite (ONOO^−^).^1^, ^12^, ^13^, ^14^, ^15^, ^16^, ^17^

Molecular hydrogen (H_2_) only quenches harmful ROS, including •OH and ONOO^−^, and permeates cell membranes; thus, it can easily target organelles, including mitochondria and nuclei. Inhaled H_2_ suppresses oxidative stress‐induced injury in several organs, such as ischaemia/reperfusion injury in the brain,^18^ liver 19 and heart,^20^ and irradiation‐induced injury in the lungs.^21^ Furthermore, continuous consumption of H_2_‐rich water protects against oxidative damage, including manifestations of oxidative stress associated with diabetes in humans,^22^ cisplatin‐induced renal injury in mice,^23^ naphthalene‐evoked acute lung injury in mice^24^ and non‐alcoholic steatohepatitis^25^, ^26^ in animal models. Thus, H_2_ may be used clinically as a safe and effective antioxidant with few side effects.

In this study, we re‐evaluated fibrotic lung lesions in D1CC mice as a model of RA‐ILD. Furthermore, we investigated the effect of H_2_ treatment in this model to evaluate its therapeutic use against chronic lung fibrosis.

## 2 MATERIALS AND METHODS

### 2.1 Production of H_2_‐rich water

H_2_‐rich water was prepared using a previously described method.^21^ Briefly, H_2_ gas (purity > 99.999%; Iwatani) was dissolved in reverse osmosis water under high pressure (0.4 MPa) to a super‐saturated level in a stainless steel tank (Unicontrols). During the preparation of H_2_‐rich water, the H_2_ concentration in the air was carefully monitored using a H_2_ sensor with an alarm for safety. The saturated water was poured into 70‐mL glass vessels equipped with an outlet line containing two ball bearings, which prevented the water from being degassed. The H_2_ concentration in the water was measured using a needle‐type H_2_ sensor (Unisense). H_2_‐rich water was approximately 80% saturated (640 μM) at 0.1 MPa at room temperature. Water obtained by degassing H_2_ from H_2_‐rich water with gentle stirring overnight was used as a control.

### 2.2 Animal treatment

**Figure 1:**
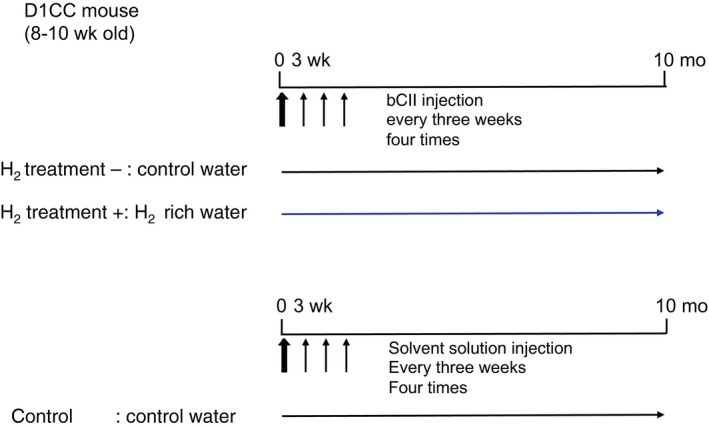
Study experimental design. D1CC mice were immunized using four 10‐µg injections of bovine type II collagen (bCII) and evaluated 10 months later. Mice consumed H_2_‐rich or control water from day 0 after the first injection until analysis. The mice were randomly assigned to three groups (n = 20 in each group): (a) control, normal water and not injected with bCII; (b) H_2_ treatment (−), normal water and injected with bCII; and (c) H_2_ treatment (+), H_2_‐rich water and injected with bCII

D1CC mouse production was described previously by Kanazawa et al,^8^ and the mice (male, 8 weeks old) used in all experiments were obtained by interbreeding six D1CC mice (three male and three female) provided by Satoshi Kanazawa. The male D1CC mice were immunized via several 10‐µg injections of bovine CII (bCII; Collagen Research Center) emulsified in an equal volume of complete Freund's adjuvant (Difco Laboratories). Mice received intradermal injections at the base of the tail, near the inguinal and axillary lymph nodes. The day of the first injection was designated Day 0. Mice received booster injections of bCII in the same location using incomplete Freund's adjuvant (Difco Laboratories) on days 21, 42 and 63. Animals consumed H_2_‐rich or control water from day 0 until analysis, and they were housed with five mice per cage under standard laboratory conditions, with food and water available ad libitum. We randomly distributed the mice into three groups (n = 20 in each group) as follows: (a) control, normal water and injected with adjuvant without bCII; (b) H_2_ treatment (−), normal water and injected with bCII; and (c) H_2_ treatment (+), H_2_‐rich water and injected with bCII. We evaluated the subgroups at 10 months after bCII injection (Figure 1). The Animal Care and Use Committee of Nippon Medical School approved the animal protocols (27‐152).

### 2.3 Evaluation of joint arthritis

D1CC mice with or without H_2_ treatment were monitored twice weekly. The clinical severity of arthritis was quantified according to a previously reported simple scoring system^8^ (Table 1) as follows: 0, no clinical symptom; 1, swelling and redness of one or two joints; 2, moderate swelling and redness of three or more joints; and 3, severe swelling and redness of the entire paw. All scores were summed to generate the total clinical score, which had a maximum value of 12 (four severely affected joints, 4 × 3).

**Table 1:** Clinical score for inflammatory arthritis

Score	Criteria
0	No effects^a^
1	Redness and swelling (one or two joints)
2	Redness and moderate swelling (three or more joints)
3	Redness, severe swelling (entire paw) and functional impairment^b^

a All joints are examined and evaluated, no differences with untreated mice are found.

b Maximal score for all four extremities is 12.

### 2.4 Sample collection

We anesthetized the mice and exsanguinated them via the abdominal aorta at specific times after bCII injection. After blood sampling and micro‐computed tomography (micro‐CT), we cannulated the trachea and removed the lungs. For each animal, the left lung (two lobes) was immediately frozen at − 80°C and later used for Western blot analyses and real‐time reverse transcription‐quantitative polymerase chain reaction (RT‐qPCR). The right lung (three lobes), which was used for microscopic analyses, was fixed for 8 hours at 4°C in 4% paraformaldehyde in 0.1 mol/L phosphate buffer (pH 7.4) at a H_2_O inflation pressure of 20 cm and then embedded in paraffin.

### 2.5 Biochemical analysis of serum

Serum surfactant protein D (SP‐D) levels were measured using a specific enzyme immunoassay kit (Yamasa) according to the manufacturer's protocol. The kit consisted of a 96‐well microtiter plate coated with monoclonal anti‐SP‐D antibodies,^27^ and colour changes were determined using a microplate reader (iMark, Bio‐Rad Laboratories, Inc) at 450 nm. The SP‐D concentration was determined by comparing the sample absorption with a standard curve.

Lipid peroxide (LPO) levels were determined using the thiobarbituric acid‐reactive substance method according to the protocol provided by Naito and Yamanaka.^28^

### 2.6 Histology and fibrosis score

We utilized haematoxylin and eosin to stain paraffin‐embedded sections for routine histological examinations and elastica Masson‐Goldner staining to investigate collagen deposition. For immunohistochemical studies, we utilized the remaining sections of the right lungs. Ashcroft scores were used to evaluate the severity of lung fibrosis (a lung lesion with inflammatory and fibrotic changes), as described previously.^29^ Briefly, we scanned eight fields of sections of the right lung (three lobes) at ×100, and we visually graded each field using scores of 0 (normal) to 8 (complete obliteration of the field by fibrosis). We defined the visual fibrosis score as the mean value of grades obtained from all inspected fields. Certain histological parameters of all samples were independently analysed by two blinded observers (N. K. and M. T.).

### 2.7 Collagen assay

The soluble collagen content of the left lung was determined using a Sircol assay (Biocolor) according to the manufacturer's instructions. Bound Sircol dye was assessed using a microplate reader (iMark) at 555 nm. Collagen standards supplied with the kit were used as assay controls.

### 2.8 Immunohistochemistry

We microwaved lung sections in DakoCytomation Target Retrieval Solution (Dako) for 10 minutes at 100°C and restricted endogenous peroxidase activity using the method of Brown et al.^30^ We immunostained sections using a Histofine Simple Stain kit (Nichirei Corporation) with rabbit polyclonal anti‐CD3 (1:400; DakoCytomation), rabbit monoclonal anti‐PAX5 (1:50; D7H5X; Cell Signaling Technology), rat monoclonal anti‐F4/80 (1:50; CI‐A3‐1; Novus Biologicals), goat polyclonal anti‐TNF‐α (1:100; Santa Cruz Biotechnology), mouse monoclonal anti‐IL‐6 (1:100; Leica Biosystems Newcastle Ltd.), transforming growth factor‐β (TGF‐β, 1:100; Santa Cruz Biotechnology), anti‐BAX (1:100; Santa Cruz Biotechnology) or mouse monoclonal anti‐8‐hydroxydeoxyguanosine (8‐OHdG) antibody (5 µg/mL; JaICA; Nikken SEIL Co., Ltd.), as previously described.^21^ We counted the 8‐OHdG‐positive cell numbers per field of view in lungs from each group (n = 6) in a blinded manner. Further, we compared the means of the positive values between each group using an image analysis system (MacScoop version 2.5, Mitani).

### 2.9 Micro‐CT

We performed micro‐CT using an LCT‐200 micro‐CT system (LaTheta; Aloka). The intensity of X‐rays in air is −1000 Hounsfield units and that in water is 0 Hounsfield units. We acquired the CT images with the X‐ray source biased at 50 kVp and 1 mA, and slices were 0.3 mm in thickness. The image size was 480 × 480 pixels, with a field of view of 48 mm at a resolution of 0.10 mm per pixel. For lung analysis, we designated areas with intensities between −400 and −200 Hounsfield units as areas of abnormally high density. The ratio of an area of abnormally high density to the whole lung field was determined using LCT‐200 system software and compared between control and bCII‐injected mice with and without H_2_ treatment.^21^

### 2.10 Western blot analysis

Western blotting was performed in each experiment according to the standard procedure. We quantified proteins using a BCA Protein Assay kit (Thermo Scientific). We homogenized frozen left lung tissues in a mammalian protein extraction reagent that contained a protein‐stabilizing cocktail (Halt Protease Inhibitor Cocktail; Thermo Scientific), 150 mmol/L NaCl, and 1 mmol/L EDTA. Samples were centrifuged at 17712*g* for 5 minutes, and supernatants containing equal amounts of protein were boiled for 5 minutes in sodium dodecyl sulphate sample buffer, separated via 10% sodium dodecyl sulphate‐polyacrylamide gel electrophoresis and transferred to polyvinylidene difluoride membranes (Thermo Scientific) using an electroblot apparatus (Invitrogen). We incubated the membranes with protein‐free T20 Tris‐buffered saline (TBS) blocking buffer (Thermo Scientific) for 1 hour at room temperature and then incubated them with antibodies against TGF‐β, TNF‐α BAX, or β‐actin at a dilution of 1:1000 (Sigma‐Aldrich) at 4°C for approximately 16 hours. The membranes were washed several times with TBS containing 0.1% Tween 20, incubated for 45 minutes with the appropriate horseradish peroxidase‐conjugated secondary antibodies (Promega), washed again with TBS containing 0.1% Tween 20 and developed using SuperSignal West Femto Luminol/Enhancer solution (Thermo Scientific). We detected the immunoreactivity on blots using an LAS‐4000 Luminescent Image Analyzer (Fujifilm) and quantified the value via densitometry using Fuji Image Gauge software (version 4.0; Fujifilm). Furthermore, the proteins were stripped from the blotting membrane after incubation for 15 minutes in Restore PLUS Western Blot Stripping Buffer (Thermo Scientific). The expression of each protein was quantified after reaction with the appropriate antibodies and expressed as a ratio to the amount of β‐actin. We compared six respective results with controls without bCII injections in six experiments (control = 1.0) and reported the results.

### 2.11 RT‐qPCR

We performed RT‐qPCR to analyse IL‐6 mRNA expression. We extracted total RNA using TRIzol reagent (Qiagen, GmbH) according to the manufacturer's instructions and utilized ready‐to‐use primer and probe sets from Applied Biosystems (Assay‐on‐Demand Gene Expression Catalog number Mm00446190m1) for IL‐6 and glyceraldehyde‐3‐phosphate dehydrogenase (GAPDH). We optimized the primer and probe concentrations for each target gene according to the manufacturer's instructions and performed PCR (2 minutes at 50°C, 10 minutes at 95°C, and 45 cycles of 15 seconds of denaturation at 95°C and 60 seconds of annealing at 60°C) using an ABI Prism 7000 Sequence Detection System (Applied Biosystems) and fluorescent TaqMan methodology. We quantified IL‐6 and GAPDH mRNA levels in triplicate for all experiments and normalized IL‐6 mRNA expression to GAPDH mRNA expression. Results were expressed relative to the standard sample (1 × standard sample = 1.0).

### 2.12 Statistical analysis

We calculated arithmetic means and standard errors of the means for each data set and applied Student's *t* test to compare paired or independent variables. We determined the statistical differences among groups using one‐way ANOVA and considered *P* < .05 to be statistically significant.

## 3 RESULTS

### 3.1 Serum SP‐D and pathological analysis of the RA‐ILD model

**Figure 2:**
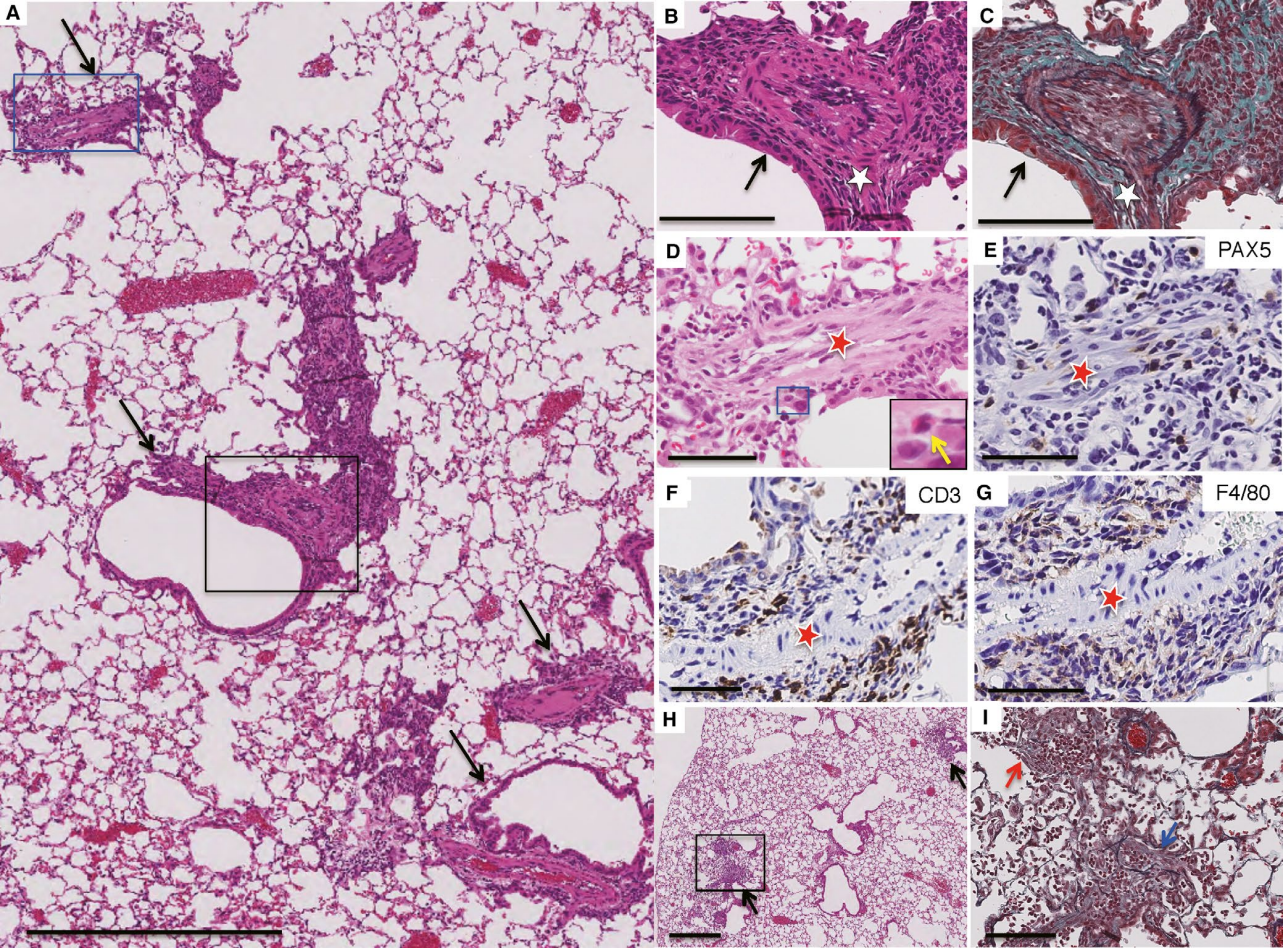
Histology of lung lesions in D1CC mice 10 months after injection with bovine type II collagen (bCII). Paraffin‐embedded lung serial sections were stained with haematoxylin and eosin (HE) (A, B, D, H) or elastica Masson‐Goldner (EMG) (C, I) or immunostained for B cells (E), T cells (F) or macrophages (G). (A) Low‐power view of a lung lesion from a D1CC mouse. Arrows denote the lymphatic distribution of inflammatory changes along with the bronchovascular bundles. (B, C) Active inflammatory lesions in the peribronchial area (white star) as high‐magnification images (HE and EMG, respectively) of the area in the black rectangle in (A). The black arrows in (B, C) point to the bronchial epithelium. (D‐G) Inflammatory cell‐infiltrated lesions in the perivascular area (red stars) in a high‐magnification image of the area in the blue rectangle in (A). The inset in (D) shows a high‐magnification image of the area in the blue rectangle in (D). Infiltrated inflammatory cells are granulocytes (yellow arrow, inset; D) and B lymphocytes (E), T lymphocytes (F) and macrophages (G), as highlighted by brown staining. (H) Infiltration of inflammatory cells in a patchy distribution in the alveolar area surrounding the bronchi (black arrows). (I) Intraluminal fibrotic change (blue arrow) with inflammatory cells observed in this high‐magnification image of the area in the black rectangle in (H). Scale bars, 500 (A, H), 100 (B, C, I) and 50 µm (D‐G)

Serum SP‐D levels were significantly increased approximately 10 months (40 weeks) after the first injection of bCII (Figure S1). Numerous inflammatory cells had infiltrated the perilymphatic stromal area of the D1CC mouse lungs, including the peribronchial (Figure 2A‐C) and perivascular (Figure 2A‐G) connective tissues, featuring a patchy distribution (Figure 2A) 10 months after the first bCII injection. The infiltrating inflammatory cells in the perivascular area were clearly granulocytes (Figure 2D), lymphocytes (B cells and T cells; Figure 2E,F) and macrophages (Figure 2G). Furthermore, inflammatory cells infiltrated the alveolar area surrounding bronchioles, which exhibited pneumocyte hyperplasia and fibrotic changes (Figure 2H,I).

### 3.2 Analyses of the RA‐ILD model with and without H_2_ treatment

**Figure 3:**
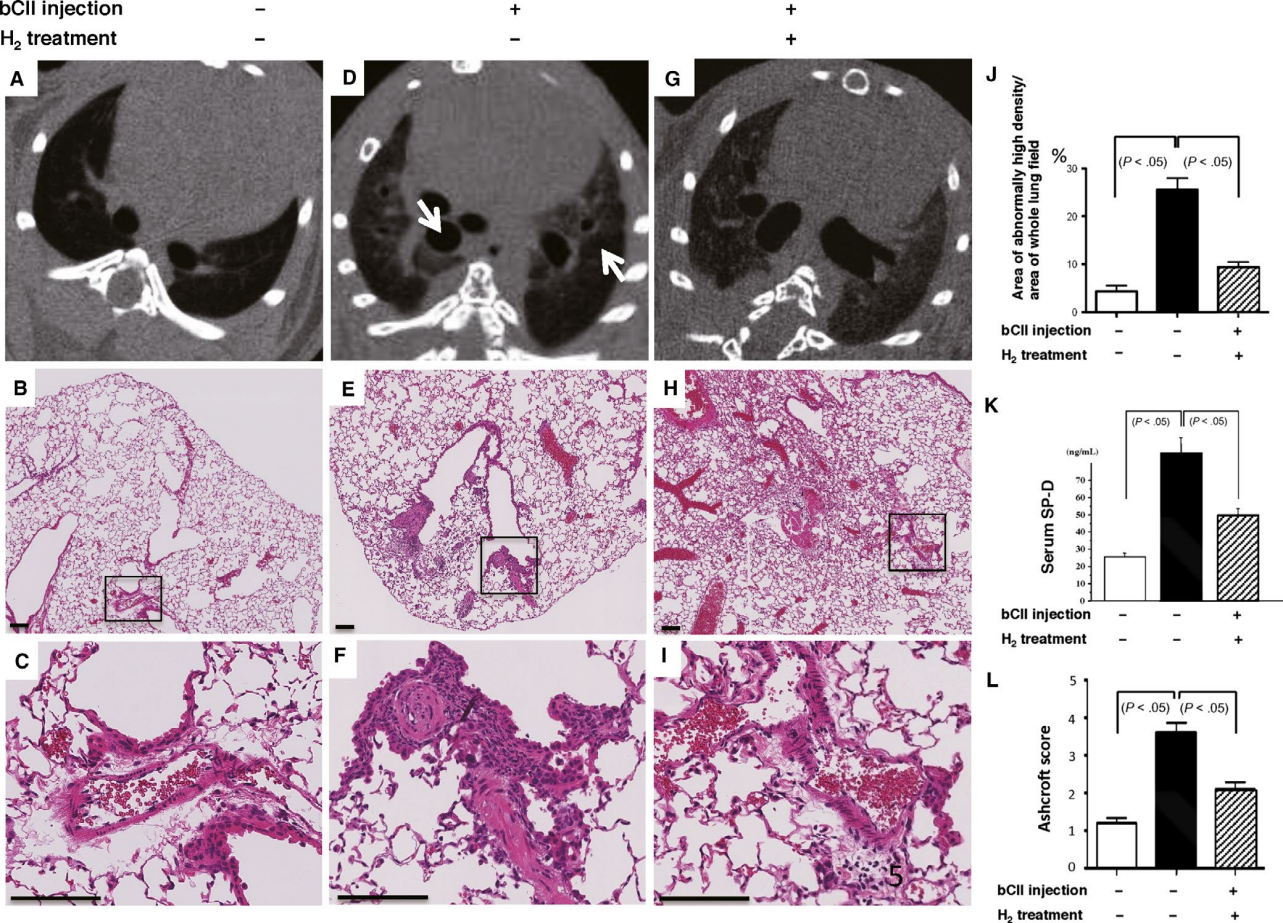
Bovine type II collagen (bCII)‐induced lung injury in D1CC mice with and without H_2_ treatment. (A, D, G) Micro‐computed tomography axial sections showing the lower thoracic areas from control and injected mice 10 months after bCII injection. bCII‐injected mice displayed a regional increase in the areas of radiopaque lesions (arrows); however, this increase was lower in mice treated with H_2_. (B, C, E, F, H, I) Representative lung regions, including a perivascular area from each group 10 months after injection (haematoxylin and eosin H&E). (C, F, I) High‐magnification images of the respective areas in black rectangles in (B, E, H). Scale bars, 100 µm. (J) Ratios of areas of abnormally high density to whole lung fields were calculated and compared for lungs from non‐injected control and injected mice with and without H_2_ treatment (n = 5). (K) Serum surfactant protein D (SP‐D) levels of non‐injected control and injected mice that consumed control or H_2_‐rich water (n = 9). (L) The severity of lung damage was evaluated using Ashcroft scores (n = 6). Data are presented as the mean ± SEM

**Figure 4:**
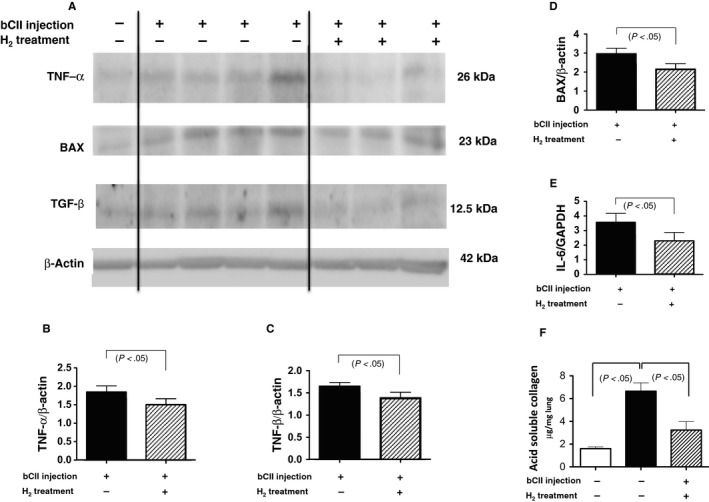
Tumour necrosis factor‐α (TNF‐α), BAX, transforming growth factor‐β (TGF‐β), interleukin‐6 (IL‐6), and soluble collagen levels after bovine type II collagen (bCII) injection with and without H_2_ treatment. (A‐D) Total lung tissue extracts from 10‐month‐old mice were subjected to Western blotting using antibodies against TNF‐α, BAX, TGF‐β and β‐actin (total loading control). (A) Representative blots from control and bCII‐injected animals without and with H_2_ treatment obtained from blotting experiments performed on the same days under the same conditions. (B‐D) In six experiments similar to those presented in (A), the amounts of each protein were quantified by densitometry and expressed relative to the amount of β‐actin in the same samples. Results are reported relative to those of six non‐injected controls (non‐injection = 1.0). Data are presented as the mean ± SEM. (E) Ratio of IL‐6 mRNA/glyceraldehyde‐3‐phosphate dehydrogenase (GAPDH) mRNA in the lungs of bCII‐injected mice as analysed using real‐time reverse transcription‐quantitative polymerase chain reaction (n = 6). Each bar represents IL‐6 mRNA levels normalized to those of GAPDH mRNA, relative to the results from six non‐injected controls (non‐injection = 1.0). Data are presented as the mean ± SEM. (F) Soluble collagen levels in the lungs of non‐injected control and injected mice with and without H_2_ treatment (n = 6) as determined using the Sircol assay. Data are presented as the mean ± SEM

Up to 8 weeks after immunization (Figure S2), we found that D1CC mice developed inflammatory arthritis with redness, swelling and fever in the joints after immunization with bCII as previously reported,^8^ whereas H_2_ treatment suppressed inflammatory arthritis development in D1CC mice induced by bCII. Ten months after the first bCII injection, we also found an increase in lung density on lung CT images (Figure 3A,D,J) and increased serum SP‐D levels (Figure S1 and Figure 3K), which were consistent with the histological findings of active alveolitis with pneumocyte hyperplasia (Figures 2 and 3B,C,E,F). H_2_ treatment protected against lung damage as evidenced by a reversal of the increases in serum SP‐D levels (Figure 3K) and lung density on CT images (Figure 3D,G,J and Figure S3) induced by bCII injection; furthermore, the Ashcroft score (Figure 3L) and soluble collagen levels (Figure 4F) were significantly decreased in DICC mice that consumed H_2_‐treated water compared with the findings in control mice.

**Figure 5:**
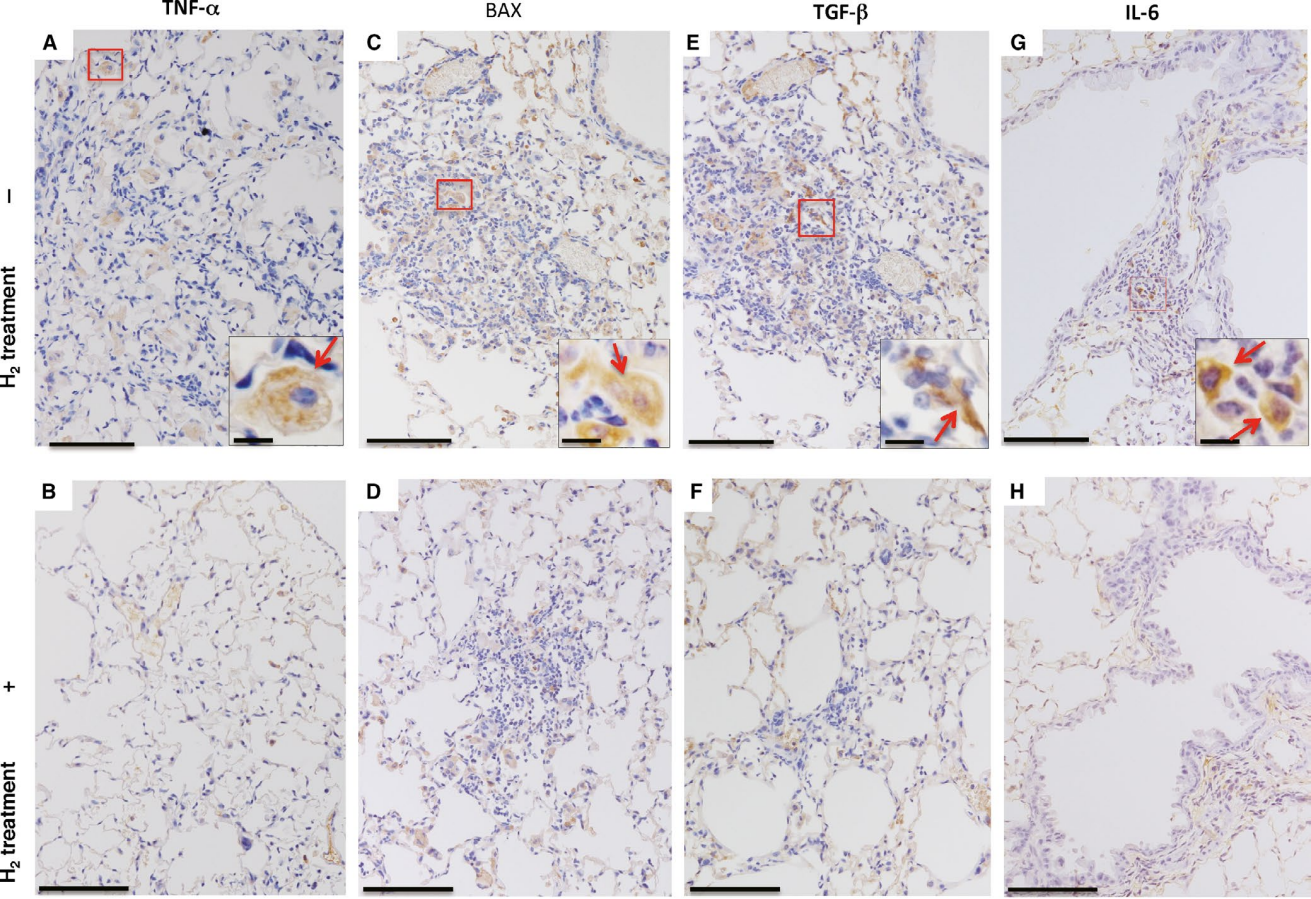
Tumour necrosis factor‐α (TNF‐α), BAX, transforming growth factor‐β (TGF‐β), interleukin‐6 (IL‐6), levels in lung lesions after bovine type II collagen (bCII) injection. Ten months after bCII injection, lung sections from each group were immunostained for TNF‐α (A), BAX (C), TGF‐β (E) and IL‐6 (G) from mice without (A, C, E, G) and with (B, D, F, H) H_2_ treatment. Insets in (A, C, E, G) are high‐magnification images of areas in the red rectangles. Positive findings for TNF‐α (A), BAX (C), TGF‐β (E) and IL‐6 (G) were observed in macrophages, pneumocytes, fibroblastic cells and plasmacytoid cells, respectively. Arrows in each inset indicate positive findings. Scale bars, 100 (main images) and 10 µm (insets, high magnification)

Western blotting and RT‐qPCR analyses revealed increased lung expression of TNF‐α, BAX, TGF‐β and IL‐6 10 months following the first bCII injection (Figure 4A‐E). H_2_ treatment protected against lung damage by significantly decreasing the magnitudes of these changes in expression (Figure 4A‐E). Consistent with these observations, immunohistochemical observation revealed that bCII treatment increased TNF‐α levels in macrophages (Figure 5A inset), BAX levels in reactive pneumocytes (Figure 5C inset), TGF‐β levels in fibroblastic cells (Figure 5E inset) and IL‐6 levels in plasmacytoid cells (Figure 5G inset) in the lung lesions in DICC mice, whereas H_2_ treatment reduced these increases (Figure 5B,D,F,H).

**Figure 6:**
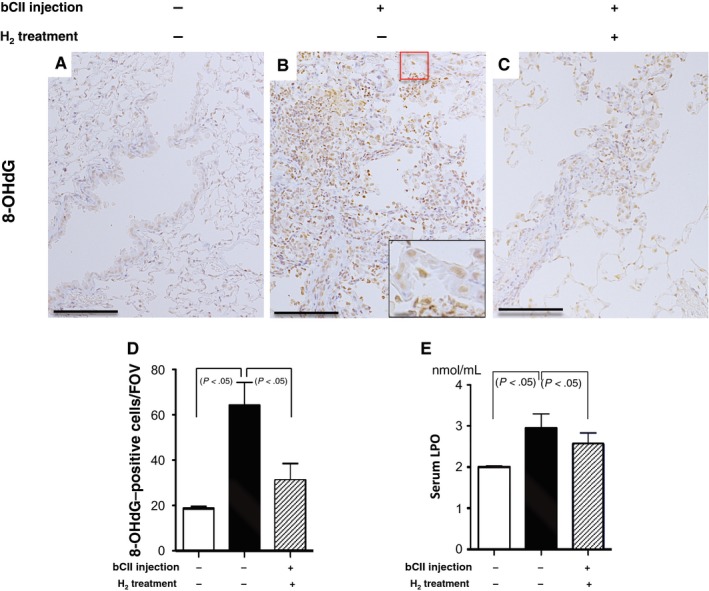
Oxidative stress in lung lesions induced by bovine type II collagen (bCII) injection with and without H_2_ treatment. (A‐C) Ten months after bCII injection, paraffin‐embedded lung sections obtained from each group were stained with 8‐hydroxydeoxyguanosine (8‐OHdG) antibody. The inset in (B) presents a high‐magnification image of the area in the red rectangle. Arrows in the inset indicate positive findings. Scale bars, 100 (main image) and 10 µm (inset, high magnification). (D) The number of 8‐OHdG‐positive cells per field of view (FOV) were counted in areas of the same size in lungs from each group (n = 6). (E) Levels of serum lipid peroxide (LPO), a marker of oxidative stress, from non‐injected control and injected mice that received H_2_ treatment or no treatment (n = 8). Data are presented as the mean ± SEM

Because ROS may be unidentified regulatory factors in RA pathogenesis,^1^, ^12^ we examined the effect of H_2_ on oxidative stress in the lungs of mice with RA based on oxidative stress markers, namely LPO levels in the serum and 8‐OHdG levels in the lungs. Serum LPO levels were significantly increased in bCII‐injected mice compared with the control levels at 10 months; however, H_2_ treatment significantly reversed these increases (Figure 6E). Consistent with these results, the number of 8‐OHdG‐positive epithelial and inflammatory cells per field of view were significantly increased in bCII‐injected mice (Figure 6B,D) compared with those in controls (Figure 6A,D), and H_2_ treatment significantly reversed these increases (Figure 6C,D).

## 4 DISCUSSION

We previously reported that D1CC transgenic mice aberrantly express major histocompatibility complex class II genes in their joints, developing RA signs such as erosive inflammatory polyarthritis, with anti‐cyclic citrullinated peptide antibody production and interstitial pneumonitis.^8^ However, we had only presented elastica and Kernechtrot staining histological data for the lungs at 6 months following CII injection.

Accordingly, in this study, we confirmed that bCII injections induce lung damage in D1CC mice (Figure 2), resulting in increased serum SP‐D levels (Figure S1 and Figure 3K) and lung density on CT images (Figure 3D,G). Furthermore, we demonstrated that mixed cellular inflammation occurs in a patchy distribution pattern (Figure 2A) in these lung lesions, mainly within the perilymphatic stromal region, such as the peribronchial (Figure 2B,C) and perivascular areas (Figure 2D‐G). We further demonstrated that a fibroinflammatory lesion develops diffusely from the proximal to the distal alveolar areas (Figure 2H,I). The cellular inflammation within the perilymphatic stromal area in this model is similar to a characteristic feature of lung lesions in patients with collagen vascular diseases, including RA.^31^

Moreover, we observed increased expression of TNF‐α, IL‐6, BAX and TGF‐β in the lungs of bCII‐injected mice compared with the findings in the lungs of control mice that consumed normal water without receiving bCII (Figure 4A). This finding is consistent with the pathological increases in the numbers of TNF‐α‐positive macrophages, IL‐6‐positive plasmacytoid cells, BAX‐positive pneumocytes and TGF‐β‐positive fibroblastic cells (Figure 5A,C,E,G). These molecules are all known to be important in the pathophysiology of RA and fibrosis in humans during inflammation, apoptosis and extracellular matrix production. Therefore, this RA‐ILD model using D1CC mice is considerably similar to human RA‐ILD and is an important animal model of this disorder.

A murine model utilizing bleomycin‐induced lung fibrosis is the most commonly used animal model of pulmonary fibrosis, and it recapitulates the general process of wound healing or repair in acute or subacute lung injury. However, it does not represent a model of chronic and progressive pulmonary damage with inflammation and fibrotic remodelling.^32^ Our RA‐ILD model in D1CC mice manifests chronic and immunologically inflammatory and progressive interstitial pneumonia with increased TNF‐α, IL‐6 and TGF‐β levels; thus, it can be considerably important as a model of RA‐ILD and chronic progressive interstitial pneumonia in humans, which is valuable for the future evaluation of biomarkers and treatments. We used this model to reveal that serum SP‐D is a suitable marker of lung damage in RA‐ILD (Figure S1 and Figure 3K), similar to serum SP‐A, SP‐D and Krebs von den Lungen‐6 as markers of idiopathic interstitial pneumonias in humans.^33^

ROS may be unidentified regulatory factors in RA pathogenesis.^1^, ^12^, ^13^, ^14^ ROS such as •OH, superoxide anions, hydrogen peroxide and hypochlorous acid are reportedly formed in the inflamed joints of patients with RA by nicotinamide adenine dinucleotide phosphate oxidase, xanthine oxidase and cytochrome P450 monooxygenase, as well as mitochondrial respiratory chain dysfunction, in chondrocytes, activated macrophages and neutrophils.^15^, ^16^, ^17^ Moreover, antioxidant therapy ameliorated arthritis in animal models; for example, α‐tocotrienol exhibited significant antioxidant and anti‐inflammatory effects on collagen‐induced arthritis in rats,^34^ and the transfection of extracellular superoxide dismutase and catalase genes ameliorated antigen‐induced arthritis in rats.^35^

H_2_ quenches only harmful ROS, such as •OH and ONOO^−^, and it can permeate cell membranes and easily target organelles, including mitochondria and nuclei.^18^ Therefore, H_2_ can be widely used as a medical treatment with good efficacy and safety and few side effects.

In this study, we illustrated that H_2_ protected against lung damage in an RA‐ILD model by decreasing serum SP‐D levels and lung density on CT images, as evidenced by histological changes in H_2_‐treated mice compared with the findings in untreated animals (Figure 3D‐L). Consistent with these results, we observed that H_2_ protects against lung damage by decreasing soluble collagen levels and TNF‐α, IL‐6, BAX and TGF‐β expression (Figures 4 and 5). These inflammatory, apoptotic and extracellular matrix molecules are important in the pathogenesis of RA and fibrosis. In addition, we found that serum LPO levels and 8‐OHdG‐positive epithelial and inflammatory cell numbers in lung lesions are significantly increased in bCII‐injected DICC mice compared with the findings in control mice that consumed normal water without receiving bCII; furthermore, we observed that these increases were significantly reversed by H_2_ treatment (Figure 6).

Together, these results indicate that oxidative stress was induced in the fibrotic lung lesions of the DICC mouse model of RA‐ILD, similar to the findings in patients with RA‐ILD. H_2_ treatment can ameliorate this oxidative stress by decreasing serum LPO levels and 8‐OHdG‐positive cell numbers and suppress inflammation and fibrosis by decreasing the expression of molecules such as TNF‐α, IL‐6 and TGF‐β in the lungs.

We previously reported a study using a nephrotoxic mouse model in which the mice consumed H_2_‐containing water. The H_2_ levels in the blood approached a concentration of several micromolar within 3 minutes of consumption, resulting in decreased oxidative stress.^23^ In humans, drinking H_2_‐rich water rapidly increases exhaled H_2_ concentrations to a maximal level of approximately 40 ppm at 10 minutes after ingestion.^36^ Therefore, continuous exposure to H_2_ via H_2_‐rich water consumption may confer, through blood circulation, protection against the oxidative state that is characteristic of RA‐ILD, although H_2_ acquired by consuming H_2_‐rich water is believed to exist for a relatively short time and at relatively low concentrations. Ingested H_2_ can spread immediately to lung cells, where it can reduce toxic ROS levels, because of the lungs' extensive blood supply and loose anatomical structure. Other studies with animal models reported similar results, that is, continuously drinking H_2_‐rich water may protect against persistent oxidative damage. These studies include research based on atherosclerosis in apolipoprotein E‐knockout mice treated for 6 months,^37^ chronic allograft nephropathy in rats treated for 5 months 38 and non‐alcoholic steatohepatitis in mice treated for 8‐16 weeks.^25^, ^26^

In this study, the RA‐ILD model is a chronic lung fibrosis model induced by the immunization of D1CC mice via several type II collagen injections; however, the precise molecular mechanisms underlying the protective effects of H_2_ remain to be elucidated. There is no satisfactory in vitro cell culture model of RA, although H_2_ has been reported to inhibit the lipopolysaccharide (LPS)‐ and IFN‐γ‐induced production of nitric oxide in macrophages in vitro by controlling signal transduction. In addition, consuming H_2_‐rich water for 2 weeks ameliorates anti‐type II collagen antibody production and LPS‐induced arthritis in mice,^39^ similar to our observations illustrating that H_2_ treatment suppressed inflammatory arthritis development in D1CC mice induced by bCII up to 8 weeks after immunization (Figure S2). Because reactive nitrogen species such as ONOO^−^ and reactive nitrogen dioxide mediate tissue damage in patients with RA through nitric oxide/nitric oxide synthase,^40^ the effect of H_2_ treatment in our model may be associated with the modulation of signal transduction by reactive nitrogen species in addition to the scavenging of ONOO^−^, which we previously reported.^18^

In conclusion, we reported that the D1CC mouse model of RA‐ILD is considerably similar to RA‐ILD in humans, and it could be valuable as a model of lung fibrosis, using specific markers such as serum SP‐D and CT to detect treatment effects. Furthermore, this model can be used in studies of the treatment and general pathophysiology of chronic interstitial pneumonias in humans. Our study revealed that H_2_ might protect against RA‐ILD by decreasing oxidative stress.

## CONFLICT OF INTEREST

The authors confirm that there are no conflicts of interest.

## AUTHOR'S CONTRIBUTIONS

Yasuhiro Terasaki, Nariaki Kokuho, Hirokazu Urushiyama, Yusuke Kajimoto, Yoko Miura, Motoyo Maruyama and Toshio Akimoto participated in animal treatment and sample collection. Nariaki Kokuho, Mika Terasakt and Satoshi Kanazawa conducted the biochemical and pathological analyses. Yasuhiro Terasaki, Satoshi Kanazawa and Shinobu Kunugi participated in the design of the study and performed the statistical analysis. Yasuhiro Terasaki, Satoshi Kanazawa, Ikuroh Ohsawa, Tsutomu Igarashi and Akira Shimizu conceived the study, participated in its design and coordination and drafted the article. All authors have read and approved the final article.

## Data Availability

The data that support the findings of this study are available from the corresponding author upon reasonable request.
