# Supplementary figures and images for: Effect of H2 treatment in a mouse model of rheumatoid arthritis‐associated interstitial lung disease
Source: J Cell Mol Med. 2019 Aug 19;23(10):7043–53. doi: 10.1111/jcmm.14603 (PMC6787460; doi:10.1111/jcmm.14603)

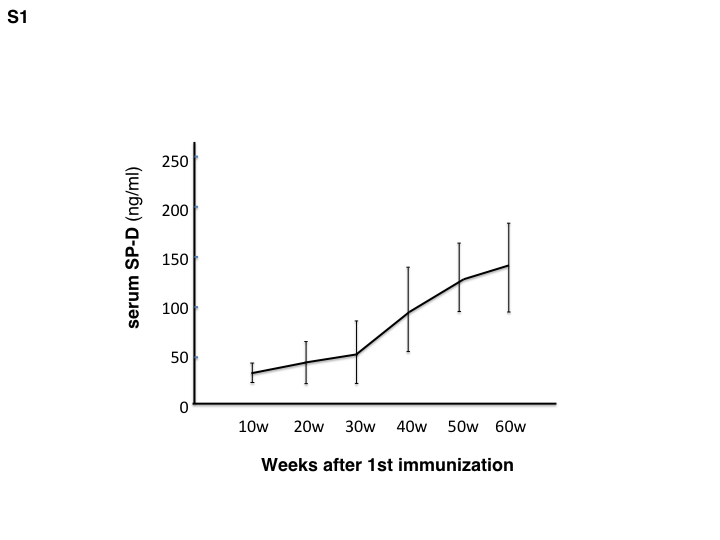

Supplement: Supplementary file 1 [file JCMM-23-7043-s001.tiff]

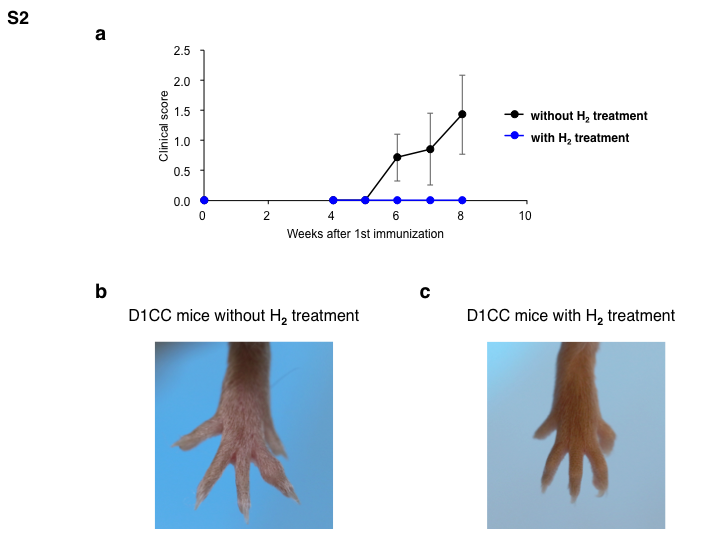

Supplement: Supplementary file 2 [file JCMM-23-7043-s002.tiff]

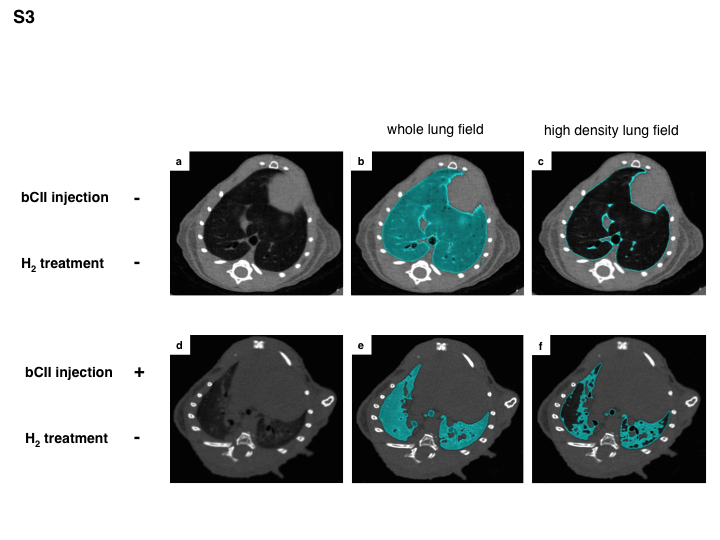

Supplement: Supplementary file 3 [file JCMM-23-7043-s003.tiff]
